# Electrochemical Micro-Immunosensor of Cubic AuPt Dendritic Nanocrystals/Ti_3_C_2_-MXenes for Exosomes Detection

**DOI:** 10.3390/mi14010138

**Published:** 2023-01-04

**Authors:** Wenpo Feng, Pingping Xu, Mei Wang, Guidan Wang, Guangda Li, Aihua Jing

**Affiliations:** 1 Medical College, Pingdingshan University, Pingdingshan 467000, China; 2 School of Medical Technology and Engineering, Henan University of Science and Technology, Luoyang 471023, China

**Keywords:** immunosensor, cubic AuPt dendritic nanocrystals, MXenes, exosomes

## Abstract

Exosomes are extracellular vesicles that exist in body circulation as intercellular message transmitters. Although the potential of tumor-derived exosomes for non-invasive cancer diagnosis is promising, the rapid detection and effective capture of exosomes remains challenging. Herein, a portable electrochemical aptasensor of cubic AuPt dendritic nanocrystals (AuPt DNs)/Ti_3_C_2_ assisted in signal amplification, and aptamer CD63 modified graphene oxide (GO) was immobilized on a screen-printed carbon electrode (SPCE) as the substrate materials for the direct capture and detection of colorectal carcinoma exosomes. Cubic AuPt DNs/Ti_3_C_2_ was synthesized according to a simple hydrothermal procedure, and the AuPt DNs/Ti_3_C_2_-Apt hybrid demonstrated an efficient recognition of exosomes. Under optimal conditions, a detection limit of down to 20 exosomes µL^−1^ was achieved with the linear range from 100 exosomes μL^−1^ to 5.0 × 10^5^ exosomes μL^−1^. The proposed immunosensor could be suitable for the analysis of exosomes and has clinical value in the early diagnosis of cancer.

## 1. Introduction

Malignant tumors are dangerous to human health due to their high morbidity and mortality. The non-invasive early diagnosis of malignant tumors has attracted attention to improve the survival rates of patients in clinical research. Exosomes, lipid-membrane-bound nanovesicles [1,2], have attracted much attention for early cancer screening technologies to provide intercellular communication with large numbers of macromolecules involving specific proteins, lipids, DNA, mRNA, and microRNAs [3]. Exosomes act as a potential tumor biomarker, which is one of the most efficient prognostic indicators for early cancer diagnosis in clinical research [4,5]. They are derived from tumor cells and mediate messages between donor cells and recipient cells, and alter immune response regulation and intracellular bioactive molecule transmission [6].

Several quantitative methods, such as enzyme-linked immunosorbent analysis [7], dynamic light scattering [8], flow cytometry [9], and nanoparticle tracking [10], have been reported for recognizing and detecting exosomes. These methods can detect exosome concentration and size as well as provide new pathways for extracellular vesicles; they can provide further utility due to their special structures and functions. However, they all have limitations such as complicated procedures, long detection time, and the difficulty in quantifying smaller exosomes. New detection techniques, such as colorimetry, fluorescence, and electrochemical techniques, have been developed for exosome identification and measurement [11,12,13]. Among these strategies, biosensors have be regarded as a valuable analytical platform in many fields, especially in the detection and monitoring of disease diagnosis and treatment, displaying the advantages of being highly selective, providing fast responses, high sensitivity [14,15], and efficient use of the analyte. The use of biosensors has attracted much attention in terms of the reorganization and detection of tumor markers, including exosomes [16]. Specifically, electrochemical immunosensors based on nanomaterials with excellent performances conjugated with aptamers for signal amplification represent an outstanding method that can quantify biomarkers in clinical diagnosis [17,18]. Nonetheless, the signal amplification of the immunosensors for exosome detection is based on the interface of the modified electrode. A high immobilizing efficiency of the biorecognition layer and a superior nanocarrier are of critical importance for increasing the sensitivity of an immunosensor. Therefore, nanomaterials that provide a rapid electric response are urgently needed.

MXenes, with a new two-dimensional (2D) transition metal carbide, layered transition metal carbide, nitride, or carbonitride, have attracted increased attention in recent years in the field of electrochemical sensors, supercapacitors, ion batteries, and liquid biopsies [19,20]. Ti_3_C_2_, as the earliest discovered MXenes material, is also the most widely used with superior properties such as excellent electron transfer, highly catalytic activity, good biocompatibility, and large surface areas. Moreover, their special nanostructure and excellent conductivity make MXenes and MXenes/metal nanoparticle hybrids potential materials for the fabrication of biosensors [21,22]. Cubic AuPt dendric nanocrystals (AuPt DNs), with excellent electrocatalytic activity and excellent biocompatibility, have been used as substrate materials for capturing antibodies in immunosensors. Wu et al. developed a biosensor based on the amino functionalization of Ti_3_C_2_ MXene for the detection of tumor biomarkers [23]. In addition, due to the abundant oxygen or hydroxyl terminated surface, Ti_3_C_2_ can interact with a majority of biomolecules in many ways, which make them unique nanobiointerface units for biosensor construction [24]. Therefore, AuPt DNs combined with Ti_3_C_2_ MXene were used as the matrix nanoprobe to selectively and reproducibly detect exosomes, and AuPt DNs showed excellent catalytic and conductive properties.

The quantitative detection of exosomes with electrochemical biosensors could be determined using antibodies as recognition molecules [25]. Aptamers, an attractive alternative to antibodies, played a key role in the determination of exosomes in recent years by binding to desired targets with excellent specificity, high stability, easy synthesis, and low cost [26]. Li et al. developed an aptasensor based on a hemin/G-quadruplex-assisted signal amplification strategy to perform the electrochemical detection of gastric cancer exosomes [27]. Tan et al. developed an aptamer-based nanotetrahedron-assisted biosensor to capture and detect hepatocellular exosomes [28]. Zhou et al. reported a microfluidic chip electrochemical biosensor based on aptamer recognition for exosome detection. A methylene blue-labeled DNA detection chain hybridizes with the CD63 aptamer, which can be determined with a detection limit of 1 × 10^6^ particles/mL. However, these aptamer-based methods are mostly nonspecific target capture methods, which make it difficult to detect tumor exosomes and nontumor exosomes. Therefore, it is urgently desirable to develop new aptasensors to recognize and detect specific exosomes (e.g., cancerous exosomes) rapidly and easily.

**Figure 1:**
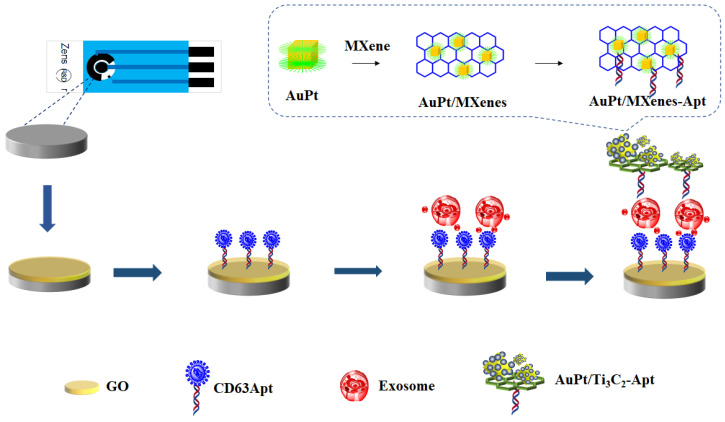
The schematic illustration of the fabrication procedure of the exosome electrochemical biosensor.

Screen printing technology was used to make on-site and point-of-care biosensors for their easy miniaturization, lower cost, disposable nature, and mass production [29]. A screen-printed carbon electrode (SPCE) is often used, with the fabrication of electrochemical immunosensors, to monitor physiological indicators in the process of diagnosis [30]. In this work, an SPCE was employed to design an electrochemical immunosensor for the efficient detection of colorectal cancer-derived exosomes (Figure 1). First, a CD63-aptamer-modified SPCE was fabricated to capture exosomes owing to its advantages such as simplicity, low cost, small size, rapid responses, and easy mass production. Moreover, the CD63-aptamer-modified cubic AuPt DNs-combined Ti_3_C_2_ MXene was selected as the matrix material to capture exosomes. The characterization of the TEM, XRD, and SEM of cubic AuPt DNs displayed the specific nanostructures of cubic AuPt DNs, which expose more active sites and, therefore, enhance the high sensitivity of the immunosensor. The optimization of detection conditions (such as the incubation time, the Apt concentration, and the concentration of KCL) for the detection of exosomes was performed. The linear range of the sensor is 100–5 × 10^5^ exosomes /µL, and the detection limit is 20 exosomes /µL (S/N = 3). The developed immunosensor was tested for exosome detection in human serum samples, which can provide a new promising method to create a miniaturized portable potentiostat working with microvolumes and for point-of-care analysis in clinical settings.

## 2. Materials and Methods

The main fabrication of the sandwich-type electrochemical biosensor is the synthesis of Ti_3_C_2_ MXenes, cubic Au/Pt DNs, and Au/Pt/Ti_3_C_2_ MXenes, which we performed as described below.

### 2.1. Reagents and Materials

Hydrogen fluoride acid was purchased from Tianjin Fuyu Fine Chemical Co., Ltd. (Tianjin, China). Ti_3_AlC_2_ was purchased from Shandong Diyan New Material Technology Co., Ltd. (Heze, China). Glycine (Gly) was from Tianjin Fengchuan Chemical Reagent Technology Co., Ltd. (Tianjin, China). N-Hydroxysuccinimide sodium salt (NHS) and 1-Ethyl-3-(3-dimethylaminopropyl) carbodiimide hydrochloride (EDC) were from Tianjin BASF chemical company (Tianjin, China). DMEM medium (high glucose), HAuCl_4_, and CD63 Apt were obtained from Sangon Biotech (Shanghai, China) Co., Ltd. Potassium ferricyanide (K_3_Fe(CN)_6_), potassium ferrocyanide (K_4_Fe(CN)_6_), and KCL were supplied by Tianjin Bodie Chemical Co., Ltd. (Tianjin, China).

### 2.2. Apparatus and Characterization

The X-ray diffraction (XRD) patterns were collected using a D8 ADVANCE X-ray diffractometer. The transmission electron microscopy (TEM) images were obtained with a JEOL 2010 transmission electron microscope. A JEOL 5160LV electron microscope was used to acquire scanning electron microscopy (SEM) images. Electrochemical detection was performed on disposable screen-printed electrodes (SPCEs) with a 3 mm diameter working area (Zensor research & development, Taiwan) connected with a CHI 660E workstation (Chenhua, Shanghai, China).

### 2.3. Cell Culture, Exosome Extraction, and Counting

HCT116 human colon cancer cell lines (HCT-1165FR) obtained from the American Type Culture Collection were maintained in MacCoy’s 5A medium (Invitrogen, Carlsbad, CA, USA) supplemented with 10% fetal bovine serum (FBS) (GIBCOBRL Laboratories, Grand Island, NY, USA) and 1% penicillomycin into DMEM in a humidified incubator at 37 °C in an atmosphere of 5% CO_2_ (Thermo Scientific, Waltham, MA, USA) [31]. When the cell concentration reached about 70%, the culture medium was replaced with a serum-free medium. After 24 h, the cell medium was collected and exosomes were separated by performing differential centrifugation. The specific steps were as follows: The culture medium (300 mL) was centrifuged at 500× *g* (g represents gravitational acceleration) for 15 min, and the supernatant was collected. The supernatant was centrifuged at 2000× *g* for 20 min and filtrated by membrane. The precipitates were then centrifuged at 3000× *g* for 15 min twice and 100,000× *g* for 90 min, before being collected and dispersed in PBS solution. Exosomes were stored at −20 °C for further use. The particle size distribution of exosomes was measured with the Malvern particle size analyzer, and the results showed that the size of exosomes was about 120 nm. The concentration of exosomes was determined with the BCA method, and the results show that the concentration of exosomes was 2.2 × 10^9^ exosomes /µL.

### 2.4. Synthesis of Ti3C2 MXenes

The multilayered Ti_3_C_2_T_X_ was synthesized using the HF etching method according to a previously reported procedure with simple modification [32,33]. A total of 1 g of Ti_3_AlC_2_ powder and 20 mL of HF (40 wt%) solution was mixed at 0 °C and stirred for 10 min. The above mixture was then stirred at 35 °C for 24 h until the reaction was completed. After that, the mixture was centrifuged at a speed of 8000 rpm and washed with deionized water until the pH was no less than 6. The precipitate Ti_3_C_2_ MXene was dispersed in deionized water by performing ultrasound and freeze-dried for 12 h.

### 2.5. Preparing Au/Pt DNs

The Au/Pt DNs were prepared according to a previously published method [34]. Firstly, 10 mL of HAuCl_4_ (0.25 mM) and CTAB (75 mM) aqueous solution were prepared in a flask, following which 0.6 mL of ice NaBH_4_ (10 mM) solution was rapidly injected, and stirred slowly for 3 h. Then, the hydrosol prepared at 1 mL was diluted to 100 mL and used as the seed solution. The colorless mixture of 0.1 mL HAuCl_4_ (10 mM), 2 mL CTAB (0.2 M), and 1.5 mL AA (ascorbic acid) (0.1 M) was diluted to 25 mL, and 0.3 mL of the prepared seed solution was added. The reaction mixture was shaken at room temperature for 8 h and a colloid containing pale purple cubic gold was obtained. Finally, AuPt DNs were prepared by adding 2 mL AA (0.1 M) and 1 mL H_2_PtCl_6_ (10 mM) to the third gold solution, successively, and washed with ultra-pure water at 60 °C for 12 h.

### 2.6. Synthesis of Au/Pt/Ti_3_C_2_ MXene

The Au/Pt/Ti_3_C_2_ MXene nanocomposite was synthesized as follows [35]: Under ultrasonic conditions, Ti_3_C_2_ MXene powder and Au/Pt powder were dispersed in DMF solution. Next, the DMF solution of Au/Pt and Ti_3_C_2_ MXene were mixed together and ultrasonicated for 3 h. The mixed solution was further stirred for 20 h. The Au/Pt/Ti_3_C_2_ MXene was collected by performing centrifugation, washed with DMF and ethanol, and then dried in vacuum at 60 °C for 12 h.

### 2.7. Preparation of Au/Pt/Ti_3_C_2_ MXene Nanoprobe

A total of 5 mg of Gly was dissolved in 5 mL of deionized water and stirred at room temperature. Then, 10 mL 1.0 mg/ mL Au/Pt-Ti_3_C_2_ MXene dispersion was added drop by drop, stirred at room temperature for 24 h, centrifuged for washing (12,000 rpm, 20 min), that is, Pt DNS-Ti_3_C_2_ MXene-Gly was prepared. After 150 μL EDC (400 mM), NHS (100 mM) and 100 μL Aptamer (-COOH) mixtures were activated at 37 °C for 1 h, 200 µL Au/Pt DNS-Ti_3_C_2_ MXene- St. Louis Gly was added to the above solution, and the reaction continued for 1 h at 37 °C. Centrifugal washing (1200, 10 min) was performed, as well as deionized water dispersion, for Au/Pt DNS-Ti_3_C_2_ MXene-GLY-Apt.

### 2.8. Preparation of Biosensor

Figure 1 shows a schematic of representation of fabrication process of the electrochemical biosensor. This article uses disposable screen-printed electrodes (SPCEs) for detection. In total, 6 μL of GO (0.25 mg/L) was dropped on the cleaned SPCE surface to gain GO/SPCE. After being dried, the electrode was immersed in EDC/NHS(100 μM/300 μM), and incubated at 37 °C for 1 h [36]. The CD63 aptamer stock solution (100 μM) was mixed with 10 mM tris(2-carboxyethyl) phosphine (TCEP, Sigma-Aldrich, St. Louis, MO, USA) for 1 h to cleave the disulfide bonds, and then 4-(2-hydroxyethyl)-1-piperazineethanesulfonic acid (HEPES, Sigma-Aldrich, St. Louis, MO, USA) buffer was used to further dilute the CD63 aptamers to 1 μM. Then, 40 μL of the prepared solution of CD63 aptamers was placed on the microelectrodes for 2 h to obtain Apt/GO/SPCEs. After incubation, the Apt/GO/SPCEs were washed with deionized (DI) water. Next, 30 μL of 1 mM 6-mercapto-1-hexanol (MCH, Sigma-Aldrich, St. Louis, MO, USA) was added to the Apt/GO/SPCEs for 15 min. To remove the excess MCH, the Apt/GO/SPCEs were washed thoroughly with DI water [37]. The Apt/GO/SPCEs were immersed in the exosomes derived from colorectal cells at certain concentrations. Then, the exosomes/Apt/GO/SPCEs were incubated with MXenes-Apt for 2 h at 37 °C.

Subsequently, the electrode that captured the exosomes was placed in a Ti_3_C_2_-MXene-Apt solution and incubated at 37 °C for 2 h. Then, the cubic AuPt DNs-Ti_3_C_2_-MXenes-Apt solution was covered on the surface to form cubic AuPt DNs-Ti_3_C_2_-MXenes-Apt/exosomes/Apt/GO/SPCEs). Finally, the cubic AuPt DNs/Ti_3_C_2_-MXenes-Apt/exosomes/Apt/GO/SPCEs electrode was ready for testing.

## 3. Results and Discussion

Electrochemical immunosensors have been a tool in the early monitoring and diagnosis of cancer. Our group demonstrated the use of this method as a label-free detection route of the cancer biomarker by dendritic tri-fan blade-like PdAuCu nanoparticles/aminefunctionalized graphene oxide [30]. In order to obtain a high sensitivity in the electrochemical immunosensors, trimetallic nanomaterials have broad applications due to their excellent properties, e.g., superior catalytic activity, better durability, and larger surface-active areas.

The major accomplishment of this work is detecting potential tumor-biomarker exosomes with nanomaterials of cubic AuPt dendritic nanocrystals/Ti_3_C_2_. Herein, the electrochemical immunosensor of cubic AuPt dendritic nanocrystals/Ti_3_C_2_-MXene are reported. The properties of the used materials are displayed first, and the characterization of the immunosensor was followed by its further application in a real sample.

### 3.1. Characterization of Ti_3_C_2_ MXenes, Ti_3_C_2_ MXenes, and Cubic AuPt DNs/Ti_3_C_2_ MXenes

**Figure 2:**
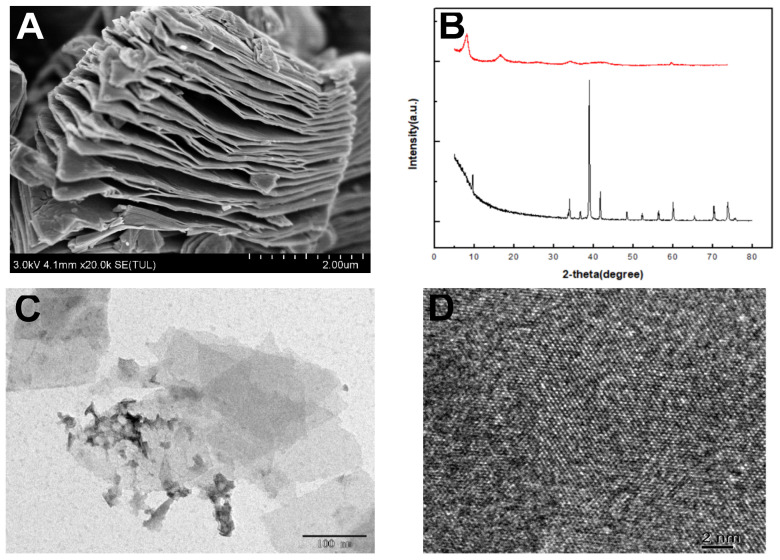
SEM (**A**), X-ray diffraction (XRD) patterns (**B**), TEM images (**C**), and HRTEM image (**D**) of the MXene Ti_3_C_2_T_x_.

The morphological structure of the as-prepared MXene-Ti_3_C_2_T_x_ and Ti_3_C_2_ MXenes was analyzed using SEM. The SEM image of Ti_3_AlC _2_ (Figure 2A) shows a multilayered structure with a typical accordion-like morphology. Figure 2C shows the morphology of the prepared MXene-Ti_3_C_2_T_x_ with thin and transparent nanoflakes stacked on each other. The layer spacing is wider than before the etching [24,25]. The structural characteristics of the as-prepared MXene-Ti_3_C_2_T_x_ and Ti_3_C_2_ MXenes composite structures were investigated by performing XRD analysis, as shown in Figure 2B. Visibly, following etching, the characteristic peak of Ti_3_AlC_2_ at 39° disappeared [26]. This proves that MXene-Ti_3_C_2_T_x_ was successfully prepared. In the pattern of Ti_3_C_2_ MXenes, the sharp peak located at ≈32° can be assigned to Ti_3_C_2_T_x_, confirming the existence of Ti_3_C_2_T_x_ support [22]. The high-resolution TEM (HRTEM) image reveals a highly crystalline lattice of the MXene basal plane without evident defects (Figure 2D), which is consistent with a previous report on MXene nanosheets prepared using LiF–HCl etchant.

**Figure 3:**
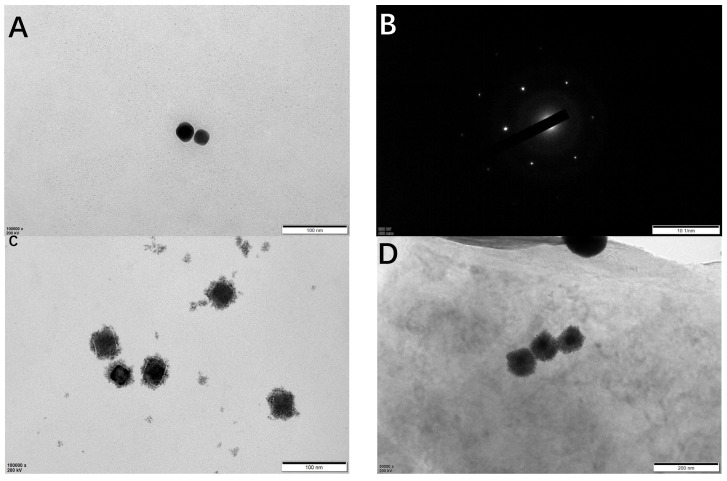
TEM images of cubic Au (**A**), diffraction pattern of cubic Au (**B**), Au@Pt DNs (**C**), and Au/Pt DNs-Ti_3_C_2_ MXene (**D**).

TEM was used to characterize Au, cubic AuPt DNs, and cubic AuPt-TI_3_C_2_ MXene. Figure 3A shows the TEM images of cubic Au, indicating that the obtained Au NPs have formed a cubic morphology with an average size of 30 nm. Figure 3B shows the diffraction pattern of cubic Au, which further confirms the successful preparation of cubic Au. Figure 3C shows the TEM image of AuPt DNs, indicating that AuPt DNs form a highly uniform dendrite form with a regular size. Figure 3D shows a visible modification of cubic AuPt DNs on the Ti_3_C_2_ MXene lamellar, which proves the successful preparation of the composite materials.

### 3.2. Electrochemical Characterizations of the Assembly Processes of the Biosensor

**Figure 4:**
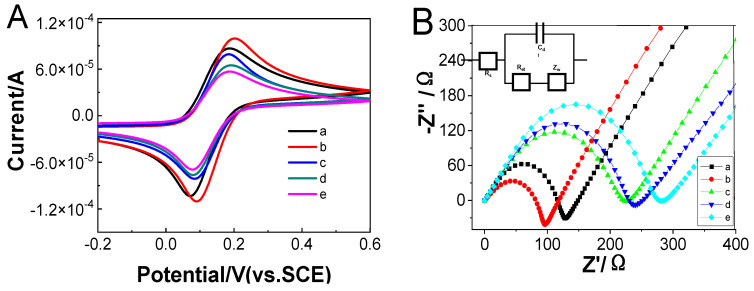
(**A**) CVs and (**B**) nyquist plots of bare (a) SPCEs, GO (b), Apt/GO (c), exosome/Apt/GO (d), cubic AuPt DNs-Ti_3_C_2_-Apt/exosome/Apt/GO (e) modified SPCEs in 0.10 M KCl containing 5.0 × 10^−3^ M K_3_[Fe(CN)_6_]/K_4_[Fe(CN)_6_].

CV and EIS were used to study the modified process of the electrochemical biosensor. Figure 4A shows the CVs of [Fe(CN)_6_]^3−^/[Fe(CN)_6_]^4−^ in PBS using the bare (curve a), GO modified (curve b), GO/Apt (curve c), GO/Apt/exosomes (curve d), and GO/Apt/exosome/Ti_3_C_2_-Apt-cubic AuPt DNs (curve e) modified SPCEs. The changes in the amperometric response correspond to the obstruction of the electron transfer kinetics of the Fe(CN)_6_^3−^/Fe(CN)_6_^4−^ probe. An electrochemical signal was detected using bare SPCEs, as shown in curve a. When GO and Apt composite materials (curves b/c) are modified on SPCEs, the peak current after modification of GO and Apt gradually decreases due to the electronic inertness. After exosomes are loaded, due to the specific binding of exosomes and aptamers, the peak current is further reduced. Due to the electronic inertness of Apt and exosomes, the electron transfer of the substrate ions on the electrode interface is hindered. Therefore, after Ti_3_C_2_ MXene modification, the peak current will also decrease. Due to the negative charge of GO, the transfer of the substrate at the electrode interface was blocked, resulting in a further reduction in the peak current.

The EIS measurement was more sensitive in monitoring the changes in the interface features in the [Fe(CN)_6_]^3−^/[Fe(CN)_6_]^4−^ probes system of the modified electrodes. Figure 4B is the EIS diagram of the modified electrode. The impedance spectrum of EIS is divided into two parts, a semicircular part and a linear part. The semicircle part represents the electron transfer process, and the diameter of the semicircle is equal to the electron transfer resistance; the linear part corresponds to the diffusion process. After the electrode surface is assembled, the resistance increases, and the obtained result corresponds to the CV diagram, which proves that the sensor is successfully assembled.

### 3.3. Optimization of the Detection Conditions

**Figure 5:**
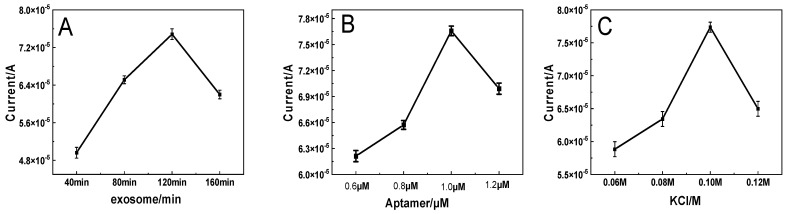
Effect of (**A**) the incubation time, (**B**) the Apt concentration, and (**C**) the concentration of KCL on the DPV response during the detection of exosomes.

In order to obtain the best analytical performance of the biosensor, the influence of the incubation time of the exosomes, the concentration of Apt, and the concentration of KCL in the substrate were detected. It can be seen from Figure 5A that the performance of this biosensor is related to the incubation time of the exosomes. The optimal incubation time of the exosomes, as shown in Figure 5A, is 120 min, when the peak current is the largest. For this reason, a 120 min incubation time was used in the following experiments.

The concentration of Apt also affects the performance of this biosensor. As shown in Figure 5B, when the concentration of Apt increased from 0.6 μM to 1.0 μM, the peak current gradually increased and did not change drastically when the Apt concentration exceeded 1.0 μM. This result shows that when the concentration of Apt is under 1.0 μM, there is not enough Apt to combine the exosome; when the concentration of Apt is 1.0 μM, Apt reaches the point of saturation on the surface of the exosome, and the ability to combine exosomes is optimal. Therefore, 1.0 μM of Atp was used for electrochemical measurement [23].

In addition, the performance of the biosensor is also related to the concentration of KCL in the substrate (Figure 5C). Before the concentration of KCL in the base solution is 0.10 M, the peak current increases, which is conducive to the transfer of electrons; however, if the concentration exceeds this, the peak current will appear to decrease, which is not conducive to the transfer of electrons. Therefore, 0.10 M KCL was used for the preparation of the biosensors.

### 3.4. The Sensitivity of the Biosensor

**Figure 6:**
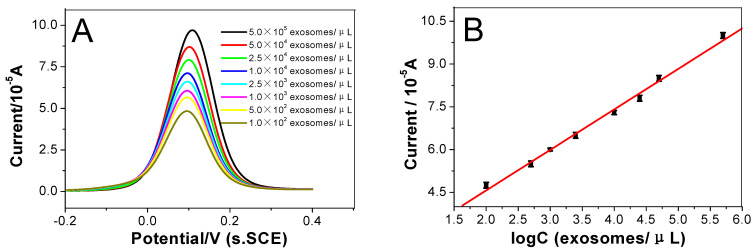
(**A**) DPV responses of the proposed immunosensor after incubation with various concentrations of exosomes in [Fe(CN)_6_]^4−/3−^. (From down to top, concentrations of exosomes are: 1.0 × 10^2^, 5.0 × 10^2^, 1.0 × 10^3^, 2.5 × 10^3^, 1.0 × 10^4^, 2.5 × 10^4^, 5.0 × 10^4^, 5.0 × 10^5^ exosomes µL^−1^); (**B**) Linear relationship between the current I of the immunosensor and logarithm of exosome concentration. Error bars represent the standard deviation, n = 5.

The designed electrochemical biosensor was used in this study strategy to detect exosomes through the DPV response curve under optimal conditions. Figure 6A illustrates the electrochemical signal intensity of the biosensor in different concentrations of colorectal cancer cell exosome solutions. These curves, from curve a to curve h in Figure 6A, represent the current responses of the as-prepared immunosensor incubated with different concentration of exosomes: 1.0 × 10^2^, 5.0 × 10^2^, 1.0 × 10^3^, 2.5 × 10^3^, 1.0 × 10^4^, 2.5 × 10^4^, 5.0 × 10^4^, and 5.0 × 10^5^ exosomes µL^−1^.

At the same time, it can be seen that the current response increases as the concentration of exosomes increased. Figure 6B shows the relationship between the logarithm of the exosome concentration and the current corresponding with what was shown in Figure 6A, and the peak current height was linear with the logarithm of the exosomes concentration in the range from 100 to 5.0 × 10^5^ exosomes μL^−1^. The linear regression equation is I = 1.726 × 10^−5^logC + 1.420 × 10^−5^ (exosomes μL^−1^), and the correlation coefficient is 0.9971. The detection limit is 20 exosomes μL^−1^ (S/N = 3). These data show that this biosensor can be used to detect exosomes with high sensitivity. Table 1 shows the comparison between the proposed electrochemical biosensor and other technologies in the electrochemical detection of exosomes. These comparisons indicated that this method is significantly better than other similar methods. This explains that the designed biosensor has good signal amplification performance.

### 3.5. Specificity and Reproducibility of the Sensor

**Figure 7:**
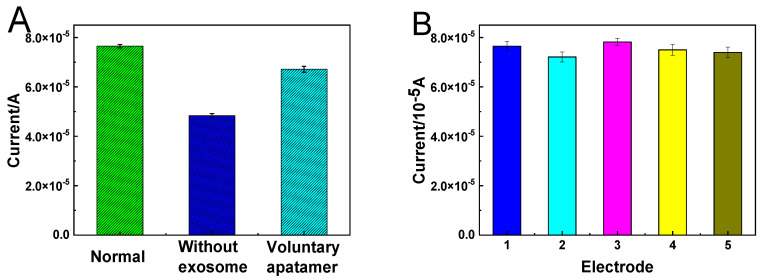
(**A**) Study of the specificity of the electrochemical biosensor toward various interferences. Error bars represent standard deviation, n = 5. The concentration of exosomes was 5.0 × 10^3^ exosomes µL^−1^. (**B**) Electrochemical responses of five immunosensor fabricated independently.

Specificity is an important parameter of electrochemical biosensors. The specificity between aptamers and exosomes was analyzed. As shown in Figure 7A, the peak current without exosomes and random Apt is lower than under normal conditions. This is because the colorectal cancer exosomes are incubated in the exosome solution, so that the exosomes are captured on the electrode; therefore, more cubic AuPt DNs-Ti_3_C_2_-Apt nanoprobes can be modified on the electrode to generate a larger current signal. At the same time, it can also be attributed to the modification of the CD63 aptamer on the electrode to capture more exosomes, so that a large number of cubic AuPt DNs-Ti_3_C_2_-Apt nanoprobes are modified on the electrode to generate a stronger current. These results show that this biosensor has good specificity. Figure 7B shows the reproducibility test of the immunosensor. Specifically, five immunosensors fabricated independently were applied to detect the target exosome (5.0 × 10^3^ exosomes µL^−1^) under the optimized conditions. The relative standard deviation (RSD) was approximately 2.8%, displaying acceptable reproducibility.

### 3.6. Detection of Cancerous Exosomes Isolated from Human Serum Samples

In order to investigate the practical application of the proposed electrochemical biosensor in biological samples, different concentrations of colorectal-derived exosomes were added to the serum for measurement. The experimental results detected are shown in Table 2, and the recoveries were in the range of 98–108%. This indicates that the developed electrochemical sensor has high accuracy in the detection of exosomes in complex samples.

## 4. Conclusions

In summary, an MXene electrochemical biosensor platform based on cubic AuPt DNs-Ti_3_C_2_ MXene was built for the detection of exosomes using an aptamer-assisted amplification strategy. The GO/SPEC interface modified by the CD63 aptamer can specifically bind to the CD63 protein on the surface of exosomes to improve their capture ability. Ti_3_C_2_ can specifically bind to a large number of CD63 aptamers and can efficiently recognize exosomes and serve as nanocarriers. The cubic AuPt DNs-Ti_3_C_2_ MXene-Apt hybrid also improves electrocatalytic activity. The fabricated electrode shows a wider linear detection range of 100~5.0 × 10^5^ exosomes μL^−1^ with a limit of detection of as low as 20 exosomes μL^−1^. In addition, this method can also be effectively applied to detect exosomes in complex serum samples. It is believed that the design of the functionalized MXene membranes expands possibilities for the implementation of 2D membrane platforms for reliable biomarker diagnosis and for the monitoring of clinical applications, such as tumor cell-promoting miRNA detection, tumor cell/exosome enrichment, and drug screening.

## Figures and Tables

**Table 1:** Comparison of the response characteristics of various modified electrodes.

Method	Detection Range (Particles μL^−1^)	Detection Limit (Particles μL^−1^)	Refs.
Electrochemical method	5 × 10^2^–5 × 10^5^	229	[38]
Electrogenerated chemiluminescence	10^2^–10^5^	30	[39]
Energy transfer	10^4^–10^9^	1.4 × 10^3^	[40]
Electrochemical method	10^3^–1.2 × 10^5^	70	[41]
Electrochemical method	1 × 10^2^–1 × 10^6^	2 × 10^2^	[42]
Electrochemical method	100–5 × 10^5^	20	This work

**Table 2:** The detection results of exosomes in serum using the biosensor.

Sample Number	Added (Log Exosome µL^−1^)	Found (Log Exosomes µL^−1^)	Recovery (%)
1	3	3.16	105
2	3.5	3.43	98
3	4	4.35	108
4	4.5	4.47	99
5	5	5.02	100

## Data Availability

The data of this work are available from the corresponding author upon reasonable request.
